# Probiotic *Lacticaseibacillus paracasei* E10 Ameliorates Dextran Sulfate Sodium-Induced Colitis by Enhancing the Intestinal Barrier and Modulating Microbiota

**DOI:** 10.3390/foods14142526

**Published:** 2025-07-18

**Authors:** Yuanyuan Dai, Ziming Lin, Xiaoyue Zhang, Yiting Wang, Yingyue Sheng, Ruonan Gao, Yan Geng, Yuzheng Xue, Yilin Ren

**Affiliations:** 1 Department of Gastroenterology, Affiliated Hospital of Jiangnan University, Wuxi 214122, China; 9862019246@jiangnan.edu.cn (Y.D.); 6222809119@stu.jiangnan.edu.cn (Z.L.); 6222809091@stu.jiangnan.edu.cn (X.Z.); 6232825025@stu.jiangnan.edu.cn (Y.W.); 9862019237@jiangnan.edu.cn (Y.S.); 6212809006@stu.jiangnan.edu.cn (R.G.); 2 School of Biotechnology, Jiangnan University, Wuxi 214122, China; 3 School of Medicine, Jiangnan University, Wuxi 214122, China; 4 School of Life Science and Health Engineering, Jiangnan University, Wuxi 214122, China

**Keywords:** probiotics, *Lacticaseibacillus paracasei* E10, DSS-induced colitis, intestinal barrier function, gut microbiota, mice

## Abstract

Inflammatory bowel disease (IBD) is a chronic gastrointestinal disorder associated with gut microbiota dysbiosis and impaired intestinal barrier function. Probiotic interventions have shown potential in alleviating intestinal inflammation and restoring microbial balance. This study explores the protective effects of *Lacticaseibacillus paracasei* (*L. paracasei*) E10 in mice. *L. paracasei* E10 demonstrated strong gastrointestinal transit tolerance, high mucosal adhesion, and probiotic properties such as hydrophobicity and aggregation ability (*p* < 0.05). The oral administration of *L. paracasei* E10 significantly alleviated colitis symptoms by reducing the disease activity index, preserving colonic architecture, increasing goblet cell density, and upregulating tight junction proteins, thereby enhancing intestinal barrier integrity. 16S rRNA sequencing revealed that *L. paracasei* E10 supplementation enriched microbial diversity, increased the abundance of Muribaculaceae, and modulated the Firmicutes/Bacteroidetes ratio, contributing to gut homeostasis. These findings indicate that *L. paracasei* E10 is a potential candidate for IBD management.

## 1. Introduction

Inflammatory bowel disease (IBD) is a gastrointestinal condition marked by non-specific inflammation, leading to diarrhea, abdominal pain, and stools that may contain mucus, pus, or blood. At present, the highest incidence of IBD is in Western Europe [1,2]. However, the incidence of IBD has shown a rapid increase in recent decades. In China, the annual growth rate for the incidence of IBD has been 1.1% for the past 30 years. In 2016, the occurrence rate of IBD in China stood at 10.04 cases every 100,000 person-years [3], with projections estimating that it will affect 1.5 million individuals by 2025. The pathogenesis of IBD involves cascade immune responses in the gut mucosa triggered by intestinal bacteria, resulting in mucosal damage in genetically susceptible individuals [4,5]. Therefore, regulating the intestinal microecological balance has become a key research focus in the treatment of IBD.

Probiotics play a role in modulating the host’s intestinal microbiome. Several lactic acid bacteria (LAB) are known probiotics that can play important physiological roles in the human body [6]. Specific LAB strains (such as *Lactobacillus acidophilus*, *Lacticaseibacillus rhamnosus*, and *Lacticaseibacillus plantarum*) are generally recognized as safe food-grade microorganisms with beneficial roles, including balancing intestinal flora, supporting immune responses, and inhibiting intestinal pathogens [7]. Preclinical studies have demonstrated that certain LAB, through the production of extracellular polysaccharides, may reduce pro-inflammatory factors and alleviate intestinal inflammation in colitis mouse models [8,9,10,11,12,13]. LAB metabolize and produce endogenous short-chain fatty acids (SCFAs) [14], including acetate, propionate, and butyrate These microbial metabolites help regulate intestinal Immunoglobulin A (IgA) levels, promote immune homeostasis, support epithelial barrier function, and contribute to colitis mitigation [15]. Therefore, LAB represent a well-established approach for managing IBD, with accumulating evidence supporting their immunomodulatory and protective roles [16].

Although LAB have shown great probiotic potential, additional research is required before they can be used as a medicine to alleviate the progress of IBD. Currently, most probiotic products are taken orally, passing through the esophagus and stomach to reach the intestines. The presence of lysozyme in saliva, stomach acid, bile, and intestinal fluid directly determines whether the bacteria can successfully survive to reach the intestines. The probiotic strain’s own ability to aggregate and adhere also helps the probiotics to colonize in the intestines. Many studies [17,18,19] have demonstrated that *L. paracasei* exhibits antioxidant properties in vivo and in vitro, reduces pro-inflammatory cytokines, and alleviates clinical symptoms in mice with enteritis [20,21]. Probiotics can interact with other microorganisms in the intestine to metabolize and produce SCFAs and linoleic acid, which can regulate host immune responses [22,23,24,25,26].

Therefore, this study aimed to evaluate the protective effects of food-derived LAB strains, particularly *L. paracasei* E10, on gut barrier function and microbiota modulation in DSS-induced colitis mouse model, with LGG serving as a positive control.

## 2. Materials and Methods

### 2.1. Bacterial Strains

Four *Lacticaseibacillus* strains were selected for this study. The experimental group included three strains: *L. paracasei* JN-1 (JN-1), *L. paracasei* JN-8 (JN-8), and *L. paracasei* E10, which were previously isolated from yogurt and vinegar in our laboratory [27]. The positive control strain, *L. rhamnosus* GG (LGG), was purchased from the ATCC (https://www.atcc.org/). All strains were cultured in MRS broth medium for 12 h without agitation and stored at −80 °C (Hopebio, Qingdao, China). These strains were chosen based on their physiological and functional characteristics, including acid tolerance, bile salt resistance, and antioxidant activity.

### 2.2. Gastrointestinal Transit Tolerance Assay

Initially, simulated gastric fluid (SGF) and simulated intestinal fluid (SIF) were prepared. *Lacticaseibacillus* cultures were inoculated into SGF at a 10% concentration and maintained at 37 °C. Three parallel experiments were conducted for each LAB strain. The OD_600_ of the fluid for bacterial survival was measured at 0 h and 3 h, as follows: Survival = [lgN1/(lgN0)] × 100%, where N1 denotes the OD_600_ of the fluid after treatment with SGF/SIF for 3 h, and N0 denotes the OD_600_ of the fluid before treatment with SGF/SIF [28,29].

### 2.3. Hydrophobicity Assay

The four *Lacticaseibacillus* species were inoculated at 1% into sterile MRS medium. The bacteria were centrifuged at 1500× *g* for 10 min following a 36 h incubation period. Then, the bacteria were washed twice before being centrifuged at 2300× *g* for 10 min. The solution’s concentration was adjusted using sterile PBS to achieve an absorbance of 1.0 at 600 nm (A_600_ = 1.0). A 2 mL bacterial solution was combined with 2 mL dimethylbenzene and vortexed for 120 s, followed by a 30 min rest at room temperature. The A_600_ of 1 mL of the aqueous phase was tested using sterile PBS as the blank control. The experiment was repeated three times independently for each strain, with triplicate samples in each repetition. The surface hydrophobic ratio of the bacteria was calculated as follows: CSH:H = [(A0 − A)/A0] × 100%, where H represents cell surface hydrophobicity, A0 represents the A_600_ value of the aqueous phase before mixing with dimethylbenzene, and A represents the A_600_ value of the aqueous phase after mixing with dimethylbenzene [30].

### 2.4. Evaluation of Bacterial Isolates for Self-Aggregation and Co-Aggregation

The four *Lacticaseibacillus* species were inoculated at 1% into sterile MRS medium. After 36 h of incubation, the *Lacticaseibacillus* bacteria were centrifuged at 1500× *g* for 10 min. The bacteria were washed twice and centrifuged at 2300× *g* for 10 min each time. The strain was diluted to achieve a standardized solution with a concentration of 1 × 10^8^ CFU/mL. A 4 mL bacterial suspension was centrifuged for 10 s, and the resulting pellet was incubated at 37 °C for 5 h. Every hour, 0.1 mL of the incubated bacterial suspension was transferred into 3.9 mL of sterile PBS solution using a pipette. The experiment was repeated three times independently for each strain, with triplicate samples in each repetition. Self-aggregation was determined using the following formula: 1 − (At/A0) × 100%, where At represents the absorbance measured after 1, 2, 3, and 4 h of incubation, and A0 denotes the initial absorbance prior to incubation [31,32].

To determine co-aggregation ability, the four *Lacticaseibacillus* species were inoculated at 1% into the sterile MRS medium. After 36 h of incubation, the *Lacticaseibacillus* bacteria were centrifuged at 1500× *g* for 10 min. The bacteria were washed twice with sterile PBS after discarding the supernatant, followed by centrifugation at 2300× *g* for 10 min. The bacteria were diluted to achieve a standard concentration of 1 × 10^8^ CFU/mL. The co-aggregation ability between the LGG, JN-1, JN-8, and E10 strains and *Escherichia coli* was tested by mixing 2 mL of bacterial suspension from each strain. Suspensions were centrifuged for 10 s and incubated at 37 °C for 5 h. The A_600_ of the bacterial suspension was recorded at 0 and 5 h. Co-aggregation (%) was determined using the following formula: [(AX + AY)/2 − A(X + Y)]/(AX + AY) * 100%, where AX and AY denote the self-aggregation. A(X + Y) denotes the A_600_ of the combined mixture [32].

### 2.5. Animal Experimental Design

Male C57BL/6 mice (7–8 weeks old, 20 ± 2 g) were acquired from Gempharmatech Co., Ltd. in Nanjing, China. Dextran sulfate sodium (DSS, CAS9011-18-1, M.W 40,000) was procured from Shanghai Macklin Biochemical Co., Ltd. (Shanghai, China) and stored at 2–8 °C. All the 36 mice were raised in the Experimental Animal Centre of the Medical College of Jiangnan University. Mice were fed a rodent chow diet. The mice ate and drank freely every day. The feeding conditions were as follows: a 12 h light/dark cycle, a temperature of 22 ± 2 °C, a humidity of 55 ± 5%, and a noise level of ≤60 dB.

After one week of adaptive accommodation, the mice were randomly divided into the following groups: the control group, DSS group, E10 group, JN-1 group, LGG group, and JN-8 group (n = 6). Mice in the same treatment group were housed together. The DSS group and the *Lacticaseibacillus* group were given 2.5% DSS (*w*/*v*, diluted in water) daily from the 7th day, while the control groups had free access to water. The control group and DSS group were then gavaged daily with 200 µL of PBS solution, and the *Lacticaseibacillus* groups were gavaged daily with 200 µL of the respective *Lacticaseibacillus* strain (1 × 10^9^ CFU) for 14 days. All mice were anesthetized through inhalation of 3% isoflurane and then euthanized using cervical dislocation on the 8th day of the experiment. The study protocol was reviewed and approved by the Jiangnan University Institutional Animal Care and Use Committee [approval no. 20220330c1440615(114)].

### 2.6. Disease Activity Index (DAI) and Colon Length Measurement

To evaluate disease progression in DSS-induced colitis, the disease activity index (DAI) was assessed daily by monitoring body weight loss, stool consistency, and the presence of occult or bloody stool [33]. Higher DAI scores indicate more severe colitis symptoms and greater intestinal inflammation [34].

### 2.7. Measurement of Colon Length

During mouse dissection, the colon was typically removed along with the cecum to facilitate accurate measurement with sterilized scissors. PBS was aspirated into a syringe and injected into the intestinal lumen to remove the contents of the colon. The true length of the colon is determined by measuring from the lower end of the cecum, excluding the cecal length from the calculation [34].

### 2.8. Examination of Mouse Colon Histology

At the end of the experiment, mouse colons were collected. The tissue was fixed in 4% paraformaldehyde for 24 h, followed by hematoxylin and eosin and periodic acid–Schiff staining [35].

### 2.9. IHC/IF of Colon of Mice

After euthanasia, the colon was surgically isolated by ligating the cecocolonic junction and distal rectum before mesenteric transection. Luminal contents were gently flushed with ice-cold PBS using a gavage needle. To comprehensively evaluate pathological alterations, the distal rectal segment was transversely sectioned and fixed in 4% paraformaldehyde (PFA) at 4 °C for 48 h. Tissues underwent graded ethanol dehydration (70–100%), xylene clearing, and triple paraffin infiltration (65 °C, 1 h/cycle). Following orientation-specific embedding, 5 µm sections were cut using a rotary microtome, heat-mounted on charged slides, and dried overnight at 60 °C for subsequent staining.

Immunohistochemistry (IHC) and immunofluorescence (IF) are two techniques used to visualize and analyze the distribution of specific proteins within cells or tissues. The process began with sectioning the tissue, followed by deparaffinization, antigen retrieval, peroxidase inactivation, and blocking to prevent non-specific binding. The primary antibody was then applied and incubated, followed by the secondary antibody and visualization with a chromogen like DAB. Finally, the slides were counterstained, mounted, and observed under a microscope.

### 2.10. Gut Microbiota Analysis

Fecal microbial community DNA was extracted with the MagPure Stool DNA KF Kit B (Magen, Shanghai, China) and quantified using a Qubit Fluorometer and a Qubit dsDNA BR Assay Kit (Invitrogen, Carlsbad, CA, USA). The PCR products were then subjected to sequencing on the Illumina MiSeq platform (BGI, Shenzhen, China). In the subsequent sequencing data processing, sequence tags exhibiting 100% similarity were clustered into the same Amplicon Sequence Variant (ASV). The ASV representative sequences were classified and analyzed using the Bayesian classifier algorithm from the Ribosomal Database Project (RDP). Alpha diversity was evaluated using MOTHUR, while Beta diversity was assessed with QIIME. The Linear Discriminant Analysis Effect Size (LEfSe) method was used to identify group-specific biomarkers and used the Phylogenetic Investigation of Communities by Reconstruction of Unobserved States (PICRUSt) tool to predict microbial functions.

### 2.11. Statistical Analysis

The data are shown as means ± standard error of the mean. The results were analyzed using unpaired two-tailed Student’s *t*-tests for the comparison between two groups with GraphPad Prism 8.4 (Graphpad Software, Inc., La Jolla, CA, USA). One-way analysis of variance (ANOVA) was used to analyze the results for the comparison between three or more groups, followed by Tukey’s multiple comparison test to determine statistical significance. *p*-values of <0.05 were deemed statistically significant.

## 3. Results

### 3.1. Gastrointestinal Tolerance and Adhesion Properties of Lacticaseibacillus Strains

**Figure 1:**
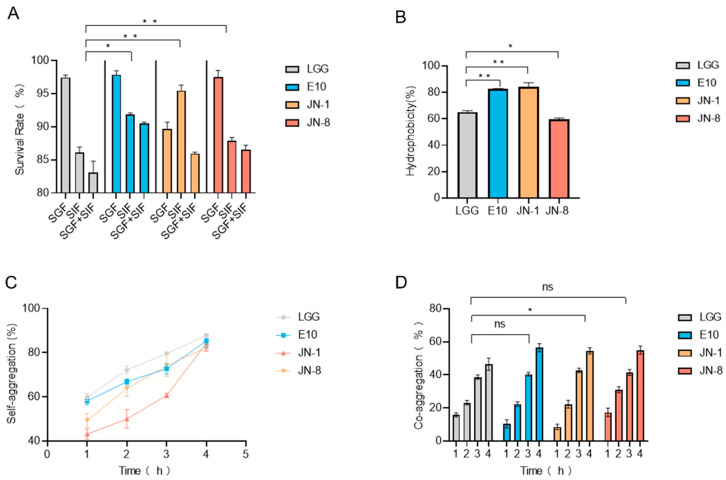
The in vitro assessments of *Lacticaseibacillus* strains. (**A**) The survival rates under simulated gastrointestinal conditions; (**B**) the hydrophobicity of *Lacticaseibacillus* strains; (**C**,**D**) the self-aggregation and co-aggregation capabilities of *Lacticaseibacillus* strains. All comparisons are made against the DSS group. * means *p* < 0.05, ** means *p* < 0.01, and ns indicates no significant difference.

The ability of *Lacticaseibacillus* strains to survive in gastrointestinal conditions is critical for their probiotic efficacy. Human gastric fluid typically has a pH of 2.0 to 3.0, with bile salt concentrations ranging from 0.03% to 0.3%. The survival capacity of four *Lacticaseibacillus* strains was assessed based on their resistance to acid and bile. Among them, *L. paracasei* E10 exhibited the highest tolerance to gastrointestinal fluid digestion (*p* < 0.05) (Figure 1A).

In addition to survival, bacterial adhesion properties, including hydrophobicity and aggregation, were evaluated. The *L. paracasei* JN-1 and E10 strains displayed the highest hydrophobicity rates, both exceeding 80%, whereas *L. paracasei* JN-8 exhibited the lowest hydrophobicity (60.26%) (*p* < 0.05) (Figure 1B). Bacterial aggregation, which can enhance colonization and resistance to environmental stressors, was also analyzed. Self-aggregation of the JN-1 and JN-8 strains ranged between 40 and 50% within the first hour, whereas LGG and E10 showed slightly higher self-aggregation (*p* > 0.05). After 4 h, the self-aggregation levels of all four strains reached approximately 80%, suggesting their strong ability to adhere to and colonize in the colon (Figure 1C).

Furthermore, the ability of these *Lacticaseibacillus* to adhere to *E. coli* was assessed. Probiotics can bind to pathogenic bacteria, facilitating their removal from the intestines through fecal excretion. Our results showed that the four *Lacticaseibacillus* strains exhibited similar adhesive properties towards *E. coli*, supporting their potential role in reducing pathogenic bacterial colonization in the gut (Figure 1D).

### 3.2. Effect of Lacticaseibacillus on Colitis

**Figure 2:**
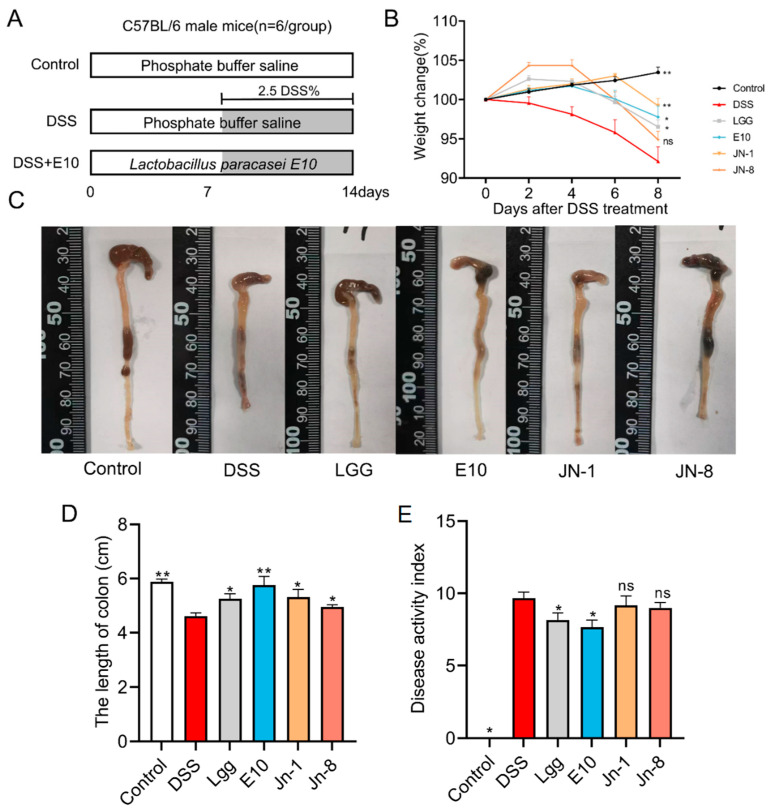
The effects of the four *Lacticaseibacillus* strains on colitis. (**A**) Experimental design and grouping; (**B**) weight changes; (**C**) representative images of mouse colons; (**D**) colon length measurements; (**E**) disease activity index (DAI). All comparisons are made against the DSS group. * means *p* < 0.05, ** means *p* < 0.01, and ns indicates no significant difference.

To investigate whether *Lacticaseibacillus* could exert anti-inflammatory properties in vivo, we performed probiotic gavage daily for mice with colitis (Figure 2A). Adding DSS to the drinking water of the mice induced symptoms of colitis, mainly manifested as diarrhea, mucous stools, fecal occult blood, gross bloody stools, weight loss, decreased activity, and poor coat condition (Figure 2B). Their body weights also showed some recovery in the E10 group (*p* < 0.05) (Figure 2B), and their colon lengths were longer than the other groups (*p* < 0.05) (Figure 2C,D). By administering different probiotics through gavage, we found that the DAI of the mice in the E10 group was the lowest (*p* < 0.05) (Figure 2E). The mice in the JN-1 group had the second-best results. The other two groups of mice performed poorly. These results indicated that *L. paracasei* E10 could contribute to recovery from intestinal inflammation in mice.

### 3.3. Protective Effects of E10 on Colitis

**Figure 3:**
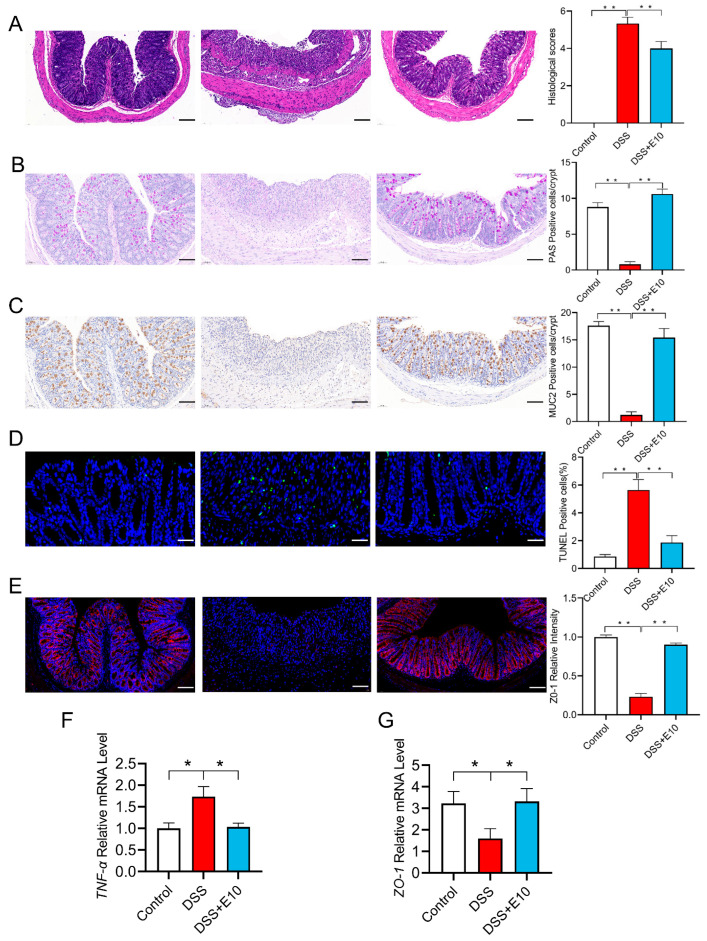
Histopathological analysis of colon tissue. (**A**) Hematoxylin and eosin stain; (**B**) AB-PAS stain; (**C**) mucin 2 immunohistochemistry (IHC) stain; (**D**) TUNEL immunofluorescence (IF) stain; (**E**) ZO-1 IF stain; (**F**,**G**) relative mRNA expression of tumor necrosis factor-α and ZO-1. All comparisons are made against the DSS group. * means *p* < 0.05, ** means *p* < 0.01, and ns indicates no significant difference. Scale bar = 100 µm.

The colonic villi of mice in the DSS group were severely damaged, and the crypt disappeared. However, the colonic tissue structure of mice after *L. paracasei* E10 intervention showed some recovery (*p* < 0.05) (Figure 3A). In the E10 gavage group, intestinal tissue exhibited fewer apoptotic cells compared to the DSS group, alongside an increase in goblet cells with neutral mucin and Zo-1 in the colon (*p* < 0.05) (Figure 3B–E). The E10 group showed a significant increase in the expression of mucin 2 and the ZO-1 (*p* < 0.05) (Figure 3F,G). This suggested that *L. paracasei* E10 has potential therapeutic value for colitis by preserving intestinal structural integrity, inhibiting apoptosis, and enhancing the mucosal barrier (*p* < 0.05). Therefore, we will further study the mechanism of *L. paracasei* E10 that relieves colitis.

### 3.4. Gut Microbiota Analysis

**Figure 4:**
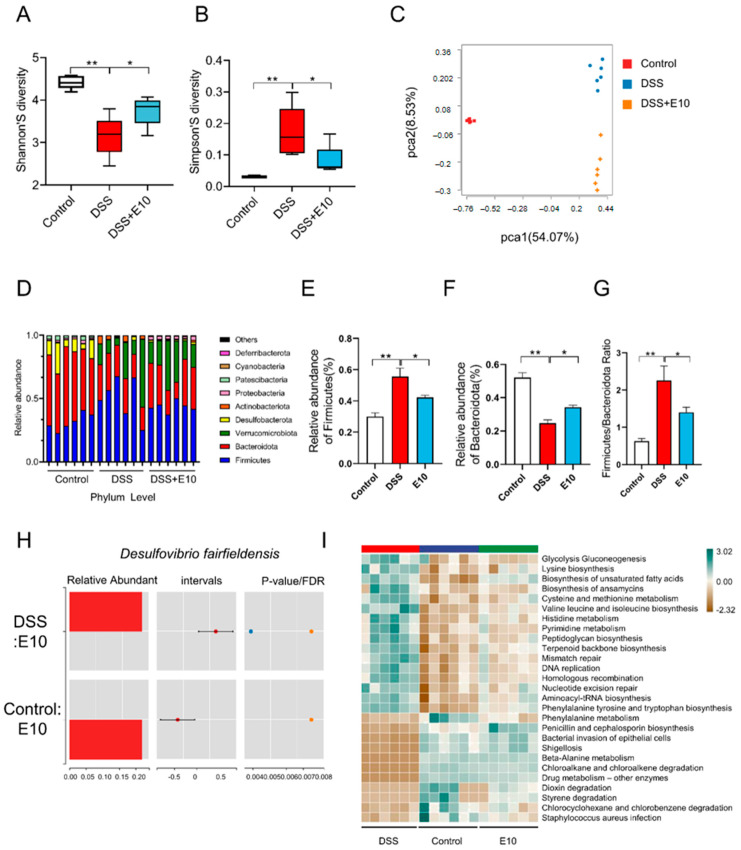
Gut microbiota composition in colitis-induced mice. (**A**) Shannon’s diversity index; (**B**) Simpson’s diversity index; (**C**) principal component analysis of gut microbiota; (**D**) stacked histogram of phylum-level species composition; (**E**,**F**) differential phylum-level species; (**G**) *Firmicutes/Bacteroidetes* ratio; (**H**) relative abundance of *Desulfovibrio fairfieldensis*; (**I**) functional prediction analysis using PICRUSt2. All comparisons are made against the DSS group. * means *p* < 0.05, ** means *p* < 0.01, and ns indicates no significant difference.

The Shannon and Simpson indices can both be used to assess the level of biodiversity in an ecosystem, including species richness and evenness, while there is a negative correlation between the two. A higher Shannon index value indicates greater biodiversity. Conversely, the smaller the value of the Simpson index, the higher the biodiversity. The 16S rRNA gene sequencing results of mouse feces showed that the Shannon index increased (Figure 4A) and the Simpson index decreased (Figure 4B) in the E10 group (*p* < 0.05). *L. paracasei* E10 enhanced the intestinal flora in mice with colitis. The composition of intestinal flora varied among the E10, control, and DSS groups (Figure 4C). The abundance of *Myxococcota*, *Elusimicrobiota,* and *Bacteroidota* was increased in the E10 group (Figure 4D). The Firmicutes/Bacteroidetes ratio is recognized as crucial for maintaining intestinal homeostasis [36]. Compared with the DSS group, the ratio of Firmicutes and Bacteroidota in the E10 group was decreased, which indicated that E10 mice may have regained the ability to maintain intestinal homeostasis (*p* < 0.05) (Figure 4E–G).

*Desulfovibrio fairfieldensis* is a sulfate-reducing bacterium and an opportunistic pathogen. Studies have shown that the bacterial outer vesicles of *D. fairfieldensis* can disrupt the intestinal epithelial barrier and activate intestinal inflammation [37]. This bacterium has also been reported to be closely associated with bacteremia in patients with cholelithiasis. The abundance of this bacterium was significantly increased in the feces of colitis mice. Gavage with E10 decreased the abundance of *Desulfovibrio* (*p* < 0.05) (Figure 4H), indicating that the probiotic *L. paracasei* E10 might inhibit the growth of conditional pathogens and consequently alleviate intestinal inflammation.

16S rRNA gene sequencing and PICRUST2 analysis reveal functional alterations in gut microbiota during inflammatory states by interpreting potential functions from microbial community composition data. The result displayed that host cells enhance energy metabolism to combat inflammatory damage; the demand for essential amino acids increases to facilitate intestinal repair and immune modulation; lipid metabolism is adaptively regulated; antioxidant capacity may decrease, affecting the synthesis of antioxidants like glutathione; the activity of specific microbial groups intensifies, potentially impacting host immunity; DNA repair and cell proliferation pathways are closely related to intestinal epithelial regeneration; the capacity for environmental pollutant degradation changes; and susceptibility to pathogen infections increases (Figure 4I). These findings provide new insights into the pathogenesis of ulcerative colitis and the development of novel therapeutic strategies.

## 4. Discussion

IBD is a chronic condition that is typically managed with medication or surgery to reduce inflammation and alleviate symptoms [38]. However, accumulating evidence suggests that probiotics with anti-inflammatory and immunomodulatory properties may offer a complementary therapeutic strategy. In this study, we identified a strain of *L. paracasei* E10 that effectively alleviated colitis symptoms in mice and enhanced gut microbiota diversity, supporting its potential role as a probiotic intervention for IBD. Probiotics with physiological characteristics such as anti-inflammatory and immune-regulating effects have been shown to alleviate colitis symptoms in mice [39,40,41]. In our study, we identified a strain of *L. paracasei* E10 that effectively alleviated colitis symptoms and enhanced gut microbiota diversity in mice.

We evaluated the in vitro physiological characteristics of a strain of *Lacticaseibacillus rhamnosus* LGG and three strains of *L. paracasei* (JN-1, JN-8 and E10), including digestion tolerance, hydrophobicity, and self- and co-aggregation with *E. coli*. SIF and SGF tolerance and self-aggregation contribute to the successful colonization of probiotics in the intestine [42]. Bacterial hydrophobicity can protect the strain from damage in the intestinal microenvironment, while co-aggregation indicates that lactobacilli can entrap pathogenic bacteria and excrete them together [43]. In these in vitro experiments, *L. paracasei* E10 had better hydrophobicity and gastrointestinal fluid tolerance, while there was no significant difference in self-aggregation and co-aggregation.

In our animal model, DSS impairs mouse intestinal epithelial cell function, compromises the intestinal barrier, and induces colitis [44,45]. We found that, under the protection of probiotics, the symptoms of colitis in mice were significantly reduced, manifested as weight gain, decreased DAI scores, and longer colon lengths. Among the tested bacteria, *L. paracasei* E10 had the greatest effect on pathological damage in this study. Subsequently, we conducted research on the physiological characteristics of *L. paracasei* E10 in colitis mice. Our study demonstrated that *L. paracasei* E10 decreased apoptotic cells and TNF-α levels in the colon tissues of colitis-afflicted mice, while enhancing ZO-1 protein levels in intestinal epithelial cells. Studies indicate that exopolysaccharides from *Lacticaseibacillus* can activate macrophages, modulate immune responses, and suppress pro-inflammatory cytokine expression [46,47].

The 16S rRNA gene sequencing result showed that the intestinal microbiota composition of DSS group mice was relatively simple, mainly dominated by the phyla Firmicutes and Bacteroidetes. *L. paracasei* E10 enhanced intestinal microbiota abundance and diversity in mice, mitigating colitis progression.

In particular, the observed effects of *L. paracasei* E10 on alleviating colitis symptoms and modulating gut microbiota composition suggest its potential as a probiotic candidate for managing inflammatory bowel disease (IBD) [39,40,41]. These results provide a rationale for future translational studies, including safety evaluation, formulation development, and possibly adjunctive use in clinical IBD management.

While this study preliminarily demonstrates the potential of *L. paracasei* E10 in alleviating colitis and modulating gut microbiota, several limitations should be acknowledged. The employed DSS-induced murine model exhibits pathophysiological differences from human IBD, necessitating further validation in more clinically relevant models or human studies. While 16S rRNA sequencing revealed compositional changes in gut microbiota, it could not characterize functional gene dynamics or metabolic profiles. Future studies should incorporate metagenomic sequencing and metabolomic analyses to provide mechanistic insights.

## 5. Conclusions

This study demonstrates that *L. paracasei* E10 exhibits promising probiotic properties and effectively alleviates DSS-induced colitis in mice. *L. paracasei* E10 demonstrates superior survival in simulated gastrointestinal conditions, strong hydrophobicity, and aggregation ability, facilitating its colonization and persistence in the gut. The administration of *L. paracasei* E10 results in reduced colitis symptoms, preserved colon architecture, increased goblet cell density, and upregulated tight junction proteins, indicating enhanced intestinal barrier integrity. Furthermore, 16S rRNA sequencing reveals that *L. paracasei* E10 supplementation enriches gut microbiota diversity, modulates the Firmicutes/Bacteroidetes ratio, and reduces the abundance of potential pathogenic bacteria like *Desulfovibrio fairfieldensis*. These findings suggest that *L. paracasei* E10 is a potential probiotic candidate for managing inflammatory bowel diseases by restoring gut homeostasis and promoting intestinal health. These findings suggest that *L. paracasei* E10 has potential as a food-deliverable probiotic strain for managing intestinal inflammation and restoring gut homeostasis.

## Data Availability

Data generated in this study can be obtained from the corresponding author upon reasonable request. The 16S rRNA gene sequencing data are available in the GEO database (https://www.ncbi.nlm.nih.gov/geo, accessed on 7 December 2024) under the specified accession number PRJNA1195230.
